# Engineering yeast with bifunctional minicellulosome and cellodextrin pathway for co-utilization of cellulose-mixed sugars

**DOI:** 10.1186/s13068-016-0554-6

**Published:** 2016-07-04

**Authors:** Li-Hai Fan, Zi-Jian Zhang, Sen Mei, Yang-Yang Lu, Mei Li, Zai-Yu Wang, Jian-Guo Yang, Shang-Tian Yang, Tian-Wei Tan

**Affiliations:** College of Life Science and Technology, Beijing University of Chemical Technology, Beijing, People’s Republic of China; Beijing Key Laboratory of Bioprocess, Beijing, People’s Republic of China; Department of Chemical and Biomolecular Engineering, The Ohio State University, Columbus, OH USA

**Keywords:** Biomass, Biofuel, Consolidated bioprocessing, *Saccharomyces cerevisiae*

## Abstract

**Background:**

Consolidated bioprocessing (CBP), integrating cellulase production, cellulose saccharification, and fermentation into one step has been widely considered as the ultimate low-cost configuration for producing second-generation fuel ethanol. However, the requirement of a microbial strain able to hydrolyze cellulosic biomass and convert the resulting sugars into high-titer ethanol limits CBP application.

**Results:**

In this work, cellulolytic yeasts were developed by engineering *Saccharomyces cerevisiae* with a heterologous cellodextrin utilization pathway and bifunctional minicellulosomes. The cell-displayed minicellulosome was two-scaffoldin derived, and contained an endoglucanase and an exoglucanase, while the intracellular cellodextrin pathway consisted of a cellodextrin transporter and a β-glucosidase, which mimicked the unique cellulose-utilization system in *Clostridium thermocellum* and allowed *S. cerevisiae* to degrade and use cellulose without glucose inhibition/repression on cellulases and mixed-sugar uptake. Consequently, only a small inoculation of the non-induced yeast cells was required to efficiently co-convert both cellulose and galactose to ethanol in a single-step co-fermentation process, achieving a high specific productivity of ~62.61 mg cellulosic ethanol/g cell·h from carboxymethyl cellulose and ~56.37 mg cellulosic ethanol/g cell·h from phosphoric acid-swollen cellulose.

**Conclusions:**

Our work provides a versatile engineering strategy for co-conversion of cellulose-mixed sugars to ethanol by *S. cerevisiae*, and the achievements in this work may further promote cellulosic biofuel production.

**Electronic supplementary material:**

The online version of this article (doi:10.1186/s13068-016-0554-6) contains supplementary material, which is available to authorized users.

## Background

Cellulosic biomass is abundant, but its degradation to fermentable glucose by a complex cocktail of cellulases with at least endoglucanase, exoglucanase and β-glucosidase is costly and hampering industrial production of cellulosic ethanol [1–4]. Consolidated bioprocessing (CBP) integrating cellulase production, cellulose saccharification, and ethanol fermentation into one step has been proposed as a cost-effective way for bioethanol production from cellulose [5]. Intensive research efforts have thus focused on engineering *Saccharomyces cerevisiae*, which has high ethanol productivity and tolerance [6, 7], but is unable to degrade cellulose, to display noncomplexed cellulase systems [8] or complexed cellulase systems (cellulosomes) [9–15].

Cellulosome is thought to have a higher activity at deconstructing cellulose than the corresponding noncomplexed system [5, 16]. However, *S. cerevisiae* had no cellodextrin transporters, so all reported minicellulosomes were designed to extracellularly hydrolyze cellulose into glucose [9–15], which could inhibit cell-displayed cellulases [17] and cause carbon catabolite repression inhibiting mixed-sugar uptake [18]. In contrast, *Clostridium thermocellum*, a natural cellulosome-producing bacterium with the highest known cellulose degradation rate [19], breaks down cellulose to mainly cellodextrins with its cellulosomal endoglucanases and exo-glucanases. Cellodextrins, including cellobiose, cellotriose and cellotetrose, are then taken up by cells through ATP-dependent transport systems and then digested into glucose by intracellular β-glucosidases and cellodextrin phosphorylases [20, 21]. This unique cellulose-utilization system in *C. thermocellum* reduces the specific inhibitory interferences by glucose on endoglucanase and exoglucanase-catalyzed reactions, and can bypass the glucose repression for simultaneously using other biomass-derived sugars.

**Fig. 1:**
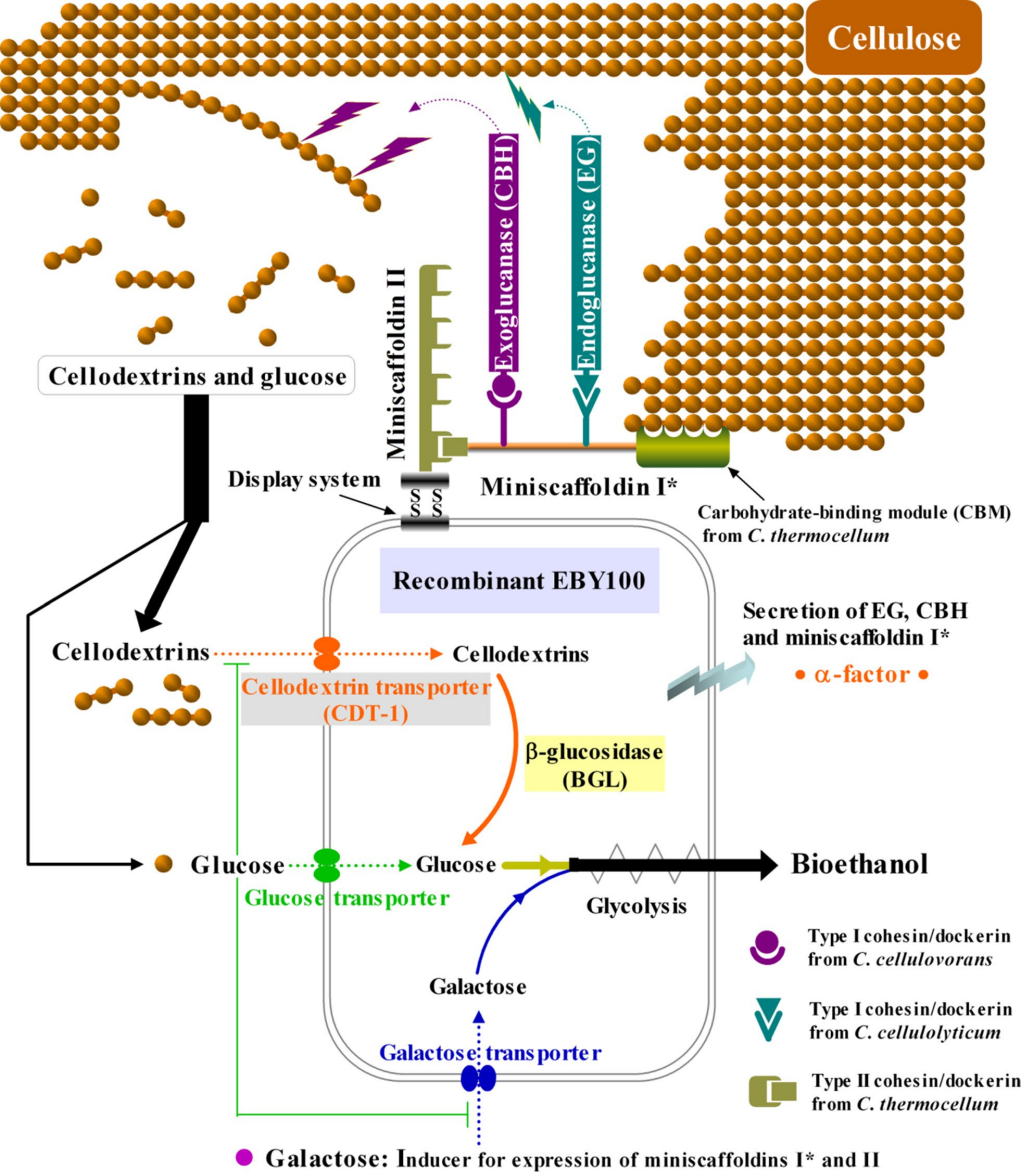
Strategy for engineering *S. cerevisiae* EBY100 to co-ferment cellulose and galactose. Endoglucanase, exoglucanase and miniscaffoldin I* were expressed as fusions to an N-terminal peptide encoding the *S. cerevisiae* α-factor secretion signal, so that they were first secreted in culture medium and then assembled extracellularly through the interactions between cohesins and dockerins. β-glucosidase and cellodextrin transporter are localized in cytoplasm and cell membrane, respectively

Yeast utilization of cellodextrins can be achieved by engineering *S. cerevisiae* with a heterologous cellodextrin transporter from *Neurospora crassa* [22]. In mixed-sugar fermentations, the engineered yeasts succeeded in bypassing glucose repression on xylose or galactose uptake, and exhibited improved ethanol production [23, 24]. In this work, a cellulose-utilization system mimicking the one in *C. thermocellum* was designed and engineered in *S. cerevisiae*. Our hypothesis was that cellulose-mixed sugars co-utilization and bioethanol production by the engineered yeast can be realized and enhanced by displaying endoglucanase and exoglucanase with a bifunctional minicellulosome on cell surface for the conversion of cellulose to cellodextrins, and employing the cellodextrin transporter from *N. crassa* for cellodextrins uptake and an intracellular β-glucosidase for cellodextrins hydrolysis to glucose, as illustrated in Fig. 1. Galactose was selected to be co-utilized with cellulose since catabolite repression of galactose by glucose is one of the best-studied eukaryotic signal integration systems [25]. Two miniscaffoldins with optimized cohesins and dockerins were displayed or secreted using galactose inducible promoters so that the efficiency of minicellulosome assembly should be affected by glucose repression, while endo- and exo-glucanases were secreted using constitutive promoters (Additional file 1: Figure S1). Yeasts with minicellulosomes were screened for enhanced cellulose hydrolysis ability. The best strain was further tested in fermentation with cellulose and galactose, as an extra carbon source and inducer for self-regulation of cell growth and minicellulosome formation, for ethanol production. Compared to other reports [9–15], the newly engineered yeasts have the advantages of higher cellulose utilization efficiency with alleviated glucose inhibition on cellulases and glucose repression on mixed-sugar uptake, achieving the highest cellulose utilization rate and specific ethanol productivity ever reported.

## Methods

### Strains and plasmids construction

*Saccharomyces cerevisiae* EBY100 (Invitrogen) was used for engineering of cellodextrin pathway and minicellulosome. *Escherichiacoli* Top10 (Biomed) was used for gene manipulation, and *E. coli* BL21 (DE3) (Biomed) was the host for expression of GFP (green fluorescent protein) fusion and enhancer [26, 27]. The genomic DNAs of *C. cellulolyticum* DSM 5812 and *C. cellulovorans* DSM 3052 were purchased from Deutsche Sammlung von Mikroorganismen und Zellkulturen (DSMZ). *Clostridium acetobutylicum* ATCC 824 was purchased from American Type Culture Collection (ATCC), while *A. niger* strain nl-1 was from Nanjing Forestry University (Nanjing). Synthetic genes of *cdt*-*1*, *gh1*-*1*, *cbh2* (codon-optimized, shown in Additional file 1), and *Enhancer* were provided by Inovogen (Beijing). Detailed descriptions of plasmids and sequences of primers are given in Additional file 1: Tables S1–S4.

### Media and culture conditions

*Escherichia coli* strains were grown in Luria-Bertani (LB) medium (1 % tryptone, 0.5 % yeast extract, 1 % NaCl) supplemented with either 100 μg/mL ampicillin or 50 μg/mL kanamycin, and were induced with 1 mM isopropyl-β-d-thiogalactopyranoside (IPTG) at 25 °C. EBY100 transformants were selected and maintained on minimal dextrose plates [0.67 % yeast nitrogen base (YNB) with ammonium sulfate and without amino acids, 2 % glucose, 1.5 % agar, appropriate supplements of Leu and Trp], and were induced in 10 mM CaCl_2_ supplied synthetic complete (SC) minimal medium (0.67 % YNB, 2 % galactose, appropriate amino acids) with an initial A_600 nm_ of 1 at 20 °C for 48 h. Cultivation of the yeasts expressing cellodextrin pathway was carried out in 96-well plate at 30 °C in SC minimal medium supplied with 2 % glucose, 1 % cellobiose, 0.5 % cellotriose, or 0.5 % cellotetrose. Cell density was periodically measured using Multiskan Spectrum (Thermo Scientific).

### Confocal laser scanning microscopy and flow cytometry analysis

*Saccharomyces cerevisiae* EBY100 transformants were cultured in SC minimal medium with 2 % glucose as the carbon source for 48 h at 25 °C. Cells were washed three times with phosphate-buffered saline (PBS, pH 7.4), and the photographs were taken with a confocal laser scanning microscope (Leica TCS SP2). The induced cells with A_600 nm_ = 1 were washed with PBS, and then incubated with anti His-tag mouse monoclonal antibody (1:100) (CWBIO) in PBS containing 0.1 % bovine serum albumin (BSA) overnight at 4 °C. After washing with PBS for two times, the cell-antibody complex was resuspended in PBS/0.1 % BSA with FITC-conjugated goat anti-mouse IgG (1:50) (CWBIO), and incubated at room temperature for 3 h. The complex was then washed and analyzed with FACSAria II (BD).

### Nanobody and enzyme assays

Enhancer and GFP (fused with docCipA) expressed *E. coli* cells were washed and resuspended in PBS (A_600 nm_ = 50). Cells were then disrupted by sonication on ice, and the cellular debris was removed by centrifugation for 10 min at 11000×*g*. SDS-PAGE was carried out on 12 % gel with prestained protein marker (10–170 kD, BioRoYee). The diluted supernatants containing GFP and Enhancer proteins were then mixed at room temperature for 2 min, and the fluorescence increase was measured with a fluorescence spectrometer at Ex = 395 nm and Em = 507 nm (F-320, Gangdong) to obtain the enhancement coefficient (α). The fluorescence from the GFP displayed on the EBY100 surface (*F*_SU_) or the GFP localized in the cell cytoplasm (*F*_IN_) were calculated using the following equations:

1 $$F_{\text{SU}} = \left( {F^{*} - F} \right)/\left( {\alpha - 1} \right)$$

2 $$F_{\text{IN}} = F - F_{\text{SU}}$$

where F* and *F* are the total fluorescence intensity of the yeast cells suspended in PBS (A_600 nm_ = 1) with and without Enhancer treatment, respectively.

After washing two times with PBS, the recombinant cells with unifunctional minicellulosomes were concentrated to A_600 nm_ = 20 in 20 mM Tris–HCl (pH 5.0) supplied with 1 % carboxymethyl cellulose (CMC) and 10 mM CaCl_2_. Viscosity reduction at 30 °C was measured periodically using an ubbelohde viscometer. The yeast cells with bifunctional minicellulosomes (A_600 nm_ = 50) were suspended in the same buffer containing 1 % CMC and 10 mM CaCl_2_, and kept at 30 °C for 20 h. The reducing sugars released were quantified by 3, 5-dinitrosalicylic acid (DNS) assay.

### Fermentation

To screen the preferred combination of endoglucanase and exoglucanase, the pre-induced yeast cells were washed twice with PBS, then concentrated to A_600 nm_ = 20 in SC minimal medium supplied with 10 mM CaCl_2_ and 1 % CMC, PASC, or Avicel. The fermentation was conducted anaerobically in rubber-stoppered glass serum bottle at 30 °C for 4 days. The yeast transformants without pre-induction were used for cellulose-galactose co-fermentation. An appropriate amount (0–25 g/L) of galactose was mixed with 1 % cellulose as the carbon source, and the growth temperature was lowered to 25 °C. Galactose was measured using D-galactose rapid kit (Megazyme), while cell density and ethanol were determined via a spectrophotometer (EU-2600, ONLAB) and gas chromatograph (GC-14C, Shimadzu), respectively.

## Results

### Functional construction of cellodextrin utilization pathway

**Fig. 2:**
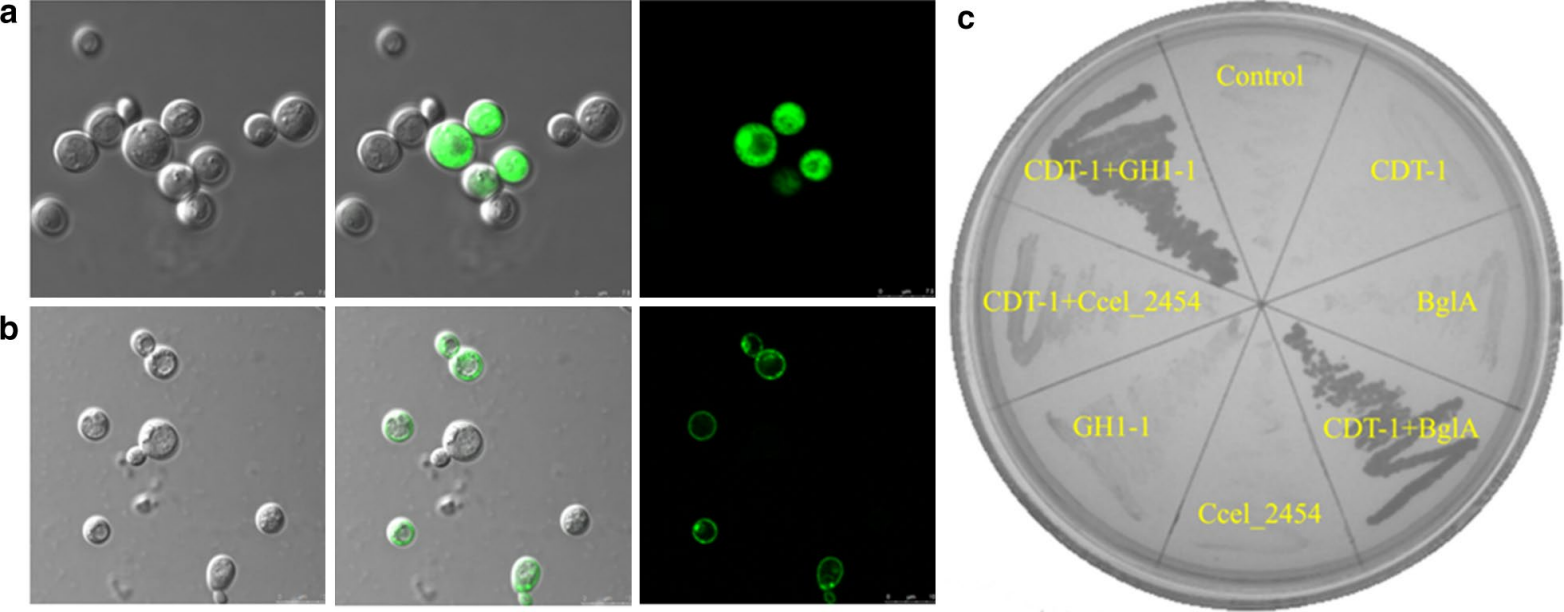
Functionality of the cellodextrin pathway in yeast. Confocal images of the EBY100 strains expressing *gfp*-fused *gh1*-*1* (**a**), and *gfp*-fused *cdt*-*1* (**b**). **c** Growth of the engineered EBY100 strains on the plate containing 1 % cellobiose as the sole carbon source. A Gly-Ser (GS) linker was introduced between GFP and GH1-1 or CDT-1 in (**a**) and (**b**). The control was transformed with pRS425. CDT-1 and β-glucosidases in (**c**) did not have GFP tag

We employed *cdt*-*1* from *N. crassa* for cellodextrins uptake. It was co-expressed with different β-glucosidases (*Ccel_2454* from *C. cellulolyticum*, *bgla* from *C. cellulovorans*, and *gh1*-*1* from *N. crassa*) to construct cellodextrin pathway in *S. cerevisiae* EBY100. As shown in Fig. 2a and b, the EBY100 strains expressing either *gfp*-fused *gh1*-*1* or *gfp*-fused *cdt*-*1* were brightly fluorescent under a confocal laser scanning microscope. The difference in GFP distribution indicated the successful localization of GH1-1 and CDT-1 in yeast cytoplasm and cell membrane, respectively. Also, Fig. 2a and b show that although GFP was detected in some cells, the rest of the population did not have GFP. The cell-to-cell heterogeneity in gene expression probably arose from fluctuations in the global gene expression machinery of the cell, which has been termed “extrinsic noise”, “global noise”, or “gene expression capacity” [28]. All three yeast transformants expressing both cellodextrin transporter and β-glucosidase were able to grow with cellobiose as the sole carbon source (Fig. 2c). They showed similar growth rates in 2 % glucose fermentation (cell density [A_600 nm_] reached ~1.45 from 0.15 after 30 h), but exhibited different growth kinetics on cellodextrins (Additional file 1: Figure S2). Among them, the strain EBY100 (*cdt*-*1, gh1*-*1*) expressing GH1-1 and CDT-1 had the highest specific growth rates and reached A_600 nm_ of 1.34, 1.21, and 1.54 from 0.15 in ~50 h with 1 % cellobiose, 0.5 % cellotriose, and 0.5 % cellotetrose as the sole carbon source, respectively.

### Facilitation of protein assembly on yeast by secretion

In this work, the N-terminals of endoglucanase, exoglucanase and miniscaffoldin I* were fused with a yeast secretion signal (α-factor) to extracellularly accomplish the minicellulosome assembly, because our earlier study [10] has suggested that direct display of the intracellularly-assembled minicellulosome may be difficult due to its large molecular mass. However, the hypothesis of using enzyme or scaffoldin secretion can facilitate cellulosome assembly on the yeast surface has never been confirmed.

**Fig. 3:**
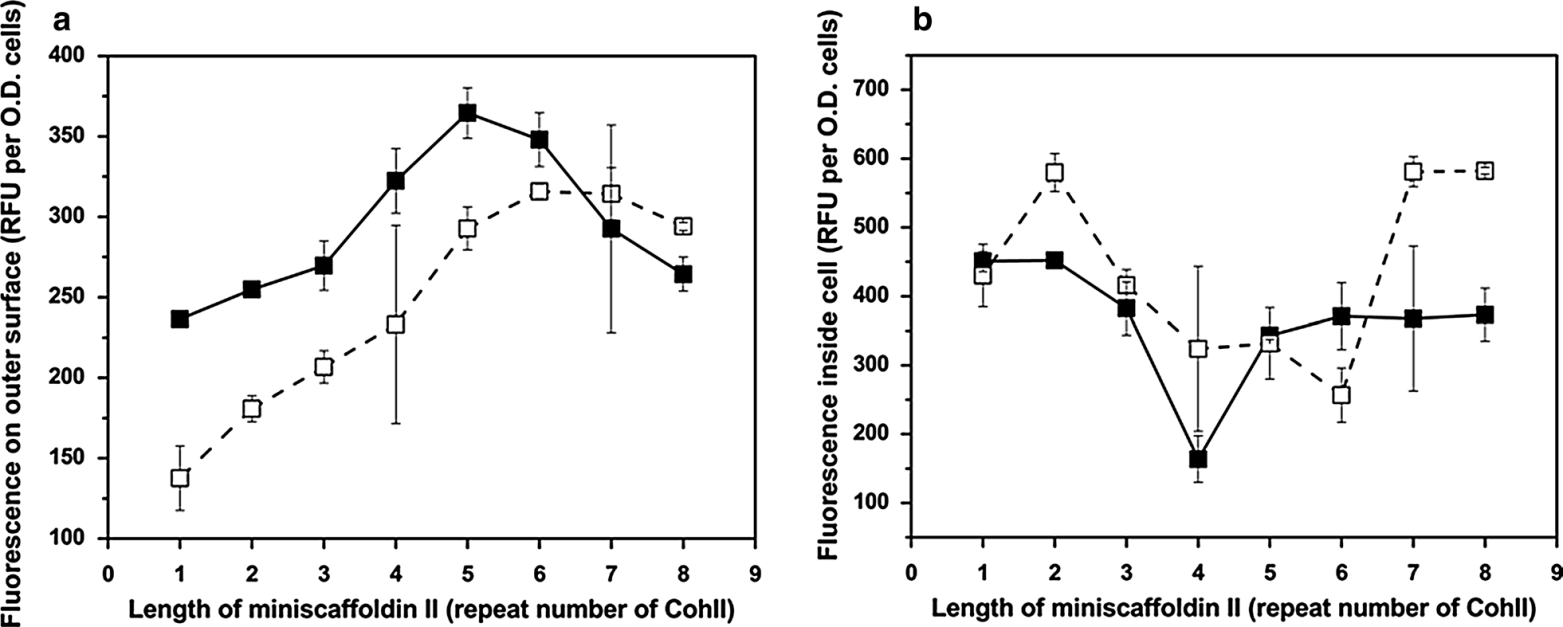
Surface assembly of GFP with or without N-terminal α-factor on the EBY100 displaying miniscaffoldin II. Fluorescence on the yeast surface (**a**), and in the cytoplasm (**b**). Assembly with α-factor (*solid* *line* with *filled* *square*), and without α-factor (*dash* *line* with *open* *square*). *gfp* was fused to *docCipA* at its C-terminus, and a GS linker was introduced between them

Here, the EBY100 displaying miniscaffoldin II and co-expressing *docCipA*-fused *gfp* (C-terminus) was thus used to investigate the impact of N-terminal α-factor on surface assembly of GFP complex. Dockerin docCipA (type II) was able to specifically bind to the type II cohesin domains (CohII) on miniscaffoldin II. To determine the amount of the surface-displayed GFP, a camelid-derived nanobody (Enhancer) [26, 27] produced in *Escherichia coli* BL21 (DE3) was applied (Additional file 1: Figure S3a). It has been reported that binding of Enhancer can facilitate improved proton extraction from the chromophore hydroxyl by His148^GFP^, thereby stabilizing the phenolate anion of the chromophore and enhancing the fluorescence intensity of GFP [26]. Here, binding of Enhancer to GFP led to an additional fluorescence increase of ~1.84-fold (Fig. S3b), thus the GFP distribution could be calculated according to Eqs. (1) and (2). Here, enhancement coefficient (α) was ~2.84. As shown in Fig. 3a, when the repeat number of CohII was less than six, the fluorescence intensity on the yeast surface increased linearly with increasing the length of miniscaffoldin II, and a raised display level of GFP usually resulted in a decrease of fluorescence inside yeast cells (Fig. 3b). The data suggested that the N-terminal α-factor was able to facilitate the formation of GFP complex on the displayed miniscaffoldin II with a fluorescence increase of >30 %. Therefore, α-factor was also fused to miniscaffoldin I*, and endo- and exo-glucanases for minicellulosome assembly.

### Functional assembly of two-scaffoldin-derived bifunctional minicellulosome

**Fig. 4:**
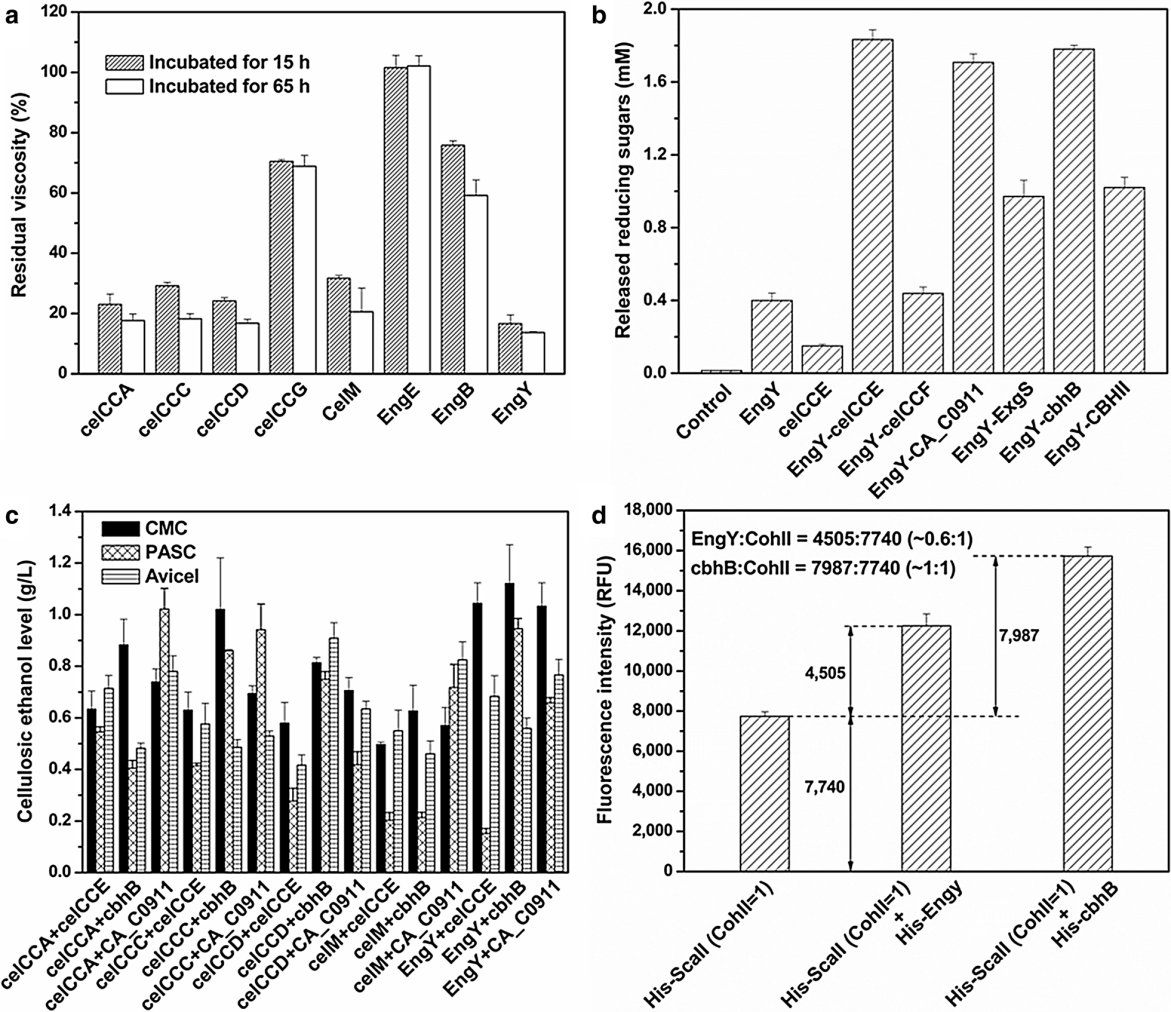
Screening of cellulases and determination of the enzyme assembly efficiency. Comparison of endoglucanases (**a**), exo-glucanases (**b**), and enzyme combinations (**c**) in various engineered yeast strains. **d** Analysis of the enzyme assembly efficiency by FACS with double-antibody staining. Control in (**b**) lacks both endoglucanase and exoglucanase. Label of *X-axis* in (**d**) indicates the fusion position of His-tag (HHHHHH) in three different EBY100 (CohII = 1, *cdt*-*1*, *gh1*-*1*, *engy*, *cbhb*). His-ScaII, His-EngY and His-cbhB mean the His-tag-fused miniscaffoldin II (ScaII, CohII = 1, C-terminus), His-tag-fused EngY (N-terminus), and His-tag-fused cbhB (N-terminus)

Eight endoglucanase genes were cloned from *C. cellulolyticum* and *C. cellulovorans* (Additional file 1: Figure S4a). These enzymes were surface assembled with miniscaffoldin I* and miniscaffoldin II (CohII = 2), respectively. Their native C-terminal dockerin domains (type I) were able to specifically bind to the cohesin domains (type I) on miniscaffoldin I*, thus unifunctional minicellulosomes could be formed after galactose induction. The decrease in the viscosity of a CMC solution was measured to evaluate the activities of the endoglucanase-associated yeasts (A_600 nm_ = 20). Figure 4a shows that EngY exhibited the highest hydrolysis ability towards CMC, resulting in a >80 % reduction in viscosity after 15 h. While celCCA, celCCC, celCCD, and celM also caused a sharp decrease in viscosity (>80 % after 65 h), followed by celCCG and EngB (<50 % after 65 h), EngE-associated EBY100 did not show any endoglucanase activity.

Then, exoglucanase genes were cloned from *C. cellulolyticum*, *C. acetobutylicum*, *C. cellulovorans*, *Aspergillus niger*, and *Trichoderma reesei* (Additional file 1: Figure S4b). They were co-expressed with *engy*, miniscaffoldin I* and miniscaffoldin II (CohII = 2), respectively, to construct bifunctional minicellulosomes on EBY100. Because of the species-specific interactions between dockerins and cohesins, the C-terminus of *CA_C0911*, *exgs*, *cbhb*, and *cbh2* were replaced or fused with dockerin *docA* from *celcca*, while the native type I dockerin domains of *celcce* and *celccf* were kept (Additional file 1: Figure S4b). To evaluate the exoglucanase activity, CMC digestion was carried out by galactose-induced EBY100 transformants with A_600 nm_ = 50. As indicated by the reducing sugars released after 20 h (Fig. 4b), celCCE, CA_C0911, and cbhB, with EngY, were obviously better in cellulose degradation, followed by CBHII, ExgS, and celCCF.

These three exoglucanase genes (*celcce*, *CA_C0911*, *cbhb*) and five endoglucanase genes (*engy*, *celcca*, *celccc*, *celccd*, *celm*) were then selected to construct bifunctional minicellulosomes on the EBY100 (*cdt*-*1, gh1*-*1*) having the cellodextrin pathway. For the endoglucanase from *C. cellulolyticum*, the C-terminal dockerin domain of the corresponding exoglucanase was replaced with dockerin *docY* from *engy* (Additional file 1: Figure S4c). The yeast transformants were first induced by galactose, then washed and concentrated to A_600 nm_ = 20, or ~6.15 g dried cell/L [23], for ethanol fermentation with CMC, phosphoric acid-swollen cellulose (PASC), or Avicel as the sole carbon source. The highest ethanol production from CMC, PASC and Avicel was 0.91–1.12 g/L by yeasts expressing the combinations of *engy* and *cbhb*, *celcca* and *CA_C0911*, and *celccd* and *cbhb*, respectively (Fig. 4c). The corresponding specific productivity was 1.54–1.90 mg cellulosic ethanol/g cell h. Three types of EBY100 (CohII = 1, *cdt*-*1*, *gh1*-*1*, *engy*, *cbhb*) were used to study the cellulase assembly efficiency. His-tag fusions in these yeasts were different (one with His-ScaII, another with His-ScaII and His-EngY, the other with His-ScaII and His-cbhB). As indicated by Additional file 1: Figure S5a and Fig. 4d, EngY:CohII reached ~0.6:1 and cbhB:CohII was ~1:1. The enzyme located at the outer fringe of the complex, which theoretically had lower steric hindrance, gathered with miniscaffoldin worked more efficiently.

### Effects of galactose, inoculum density and miniscaffoldin length

**Fig. 5:**
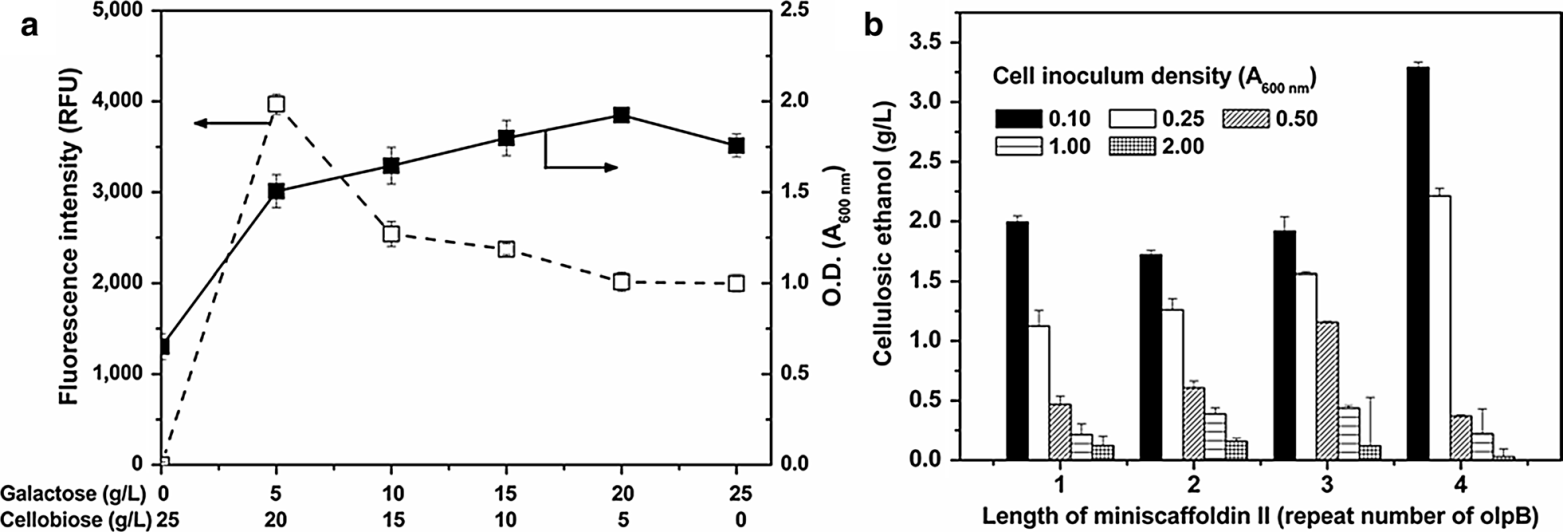
Optimization of minicellulosome assembly and cellulosic ethanol production. **a** Effects of galactose concentration on miniscaffoldin II display and cell growth. The EBY100 co-expressing bifunctional minicellulosome (CohII = 4, *engy*, *cbhb*) and cellodextrin pathway (*cdt*-*1*, *gh1*-*1*) was employed. The His-tag-fused to the C-terminus of miniscaffoldin II was double-antibody stained. **b** Effects of cell inoculum density and miniscaffoldin II length on CMC-galactose co-fermentation. The EBY100 strains co-expressing *cdt*-*1*, *gh1*-*1*, miniscaffoldin I*, miniscaffoldin II (CohII = 1–4), *engy*, and *cbhb* were used

To investigate the effects of galactose on cell growth and display efficiency of miniscaffoldin II (CohII = 4) on the EBY100 (*cdt*-*1*, *gh1*-*1, engy, cbhb*) co-expressing the cellodextrin pathway (*cdt*-*1*, *gh1*-*1*) and bifunctional minicellulosome (*engy*, *cbhb*), cells were cultured in the galactose/cellobiose mixture at various ratios. The inoculum density of EBY100 transformants was adjusted to A_600 nm_ = 0.1, and FACS was applied for the detection of miniscaffoldin II (Additional file 1: Fig. S5b). As shown in Fig. 5a, the highest fluorescence intensity of 4200 was obtained with 5 g/L galactose and 20 g/L cellobiose, while the highest cell density (A_600 nm_ = ~1.9 after 2 days) was obtained with 20 g/L galactose and 5 g/L cellobiose. Interestingly, although hydrolytic cleavage of cellodextrins through β-glucosidase could not provide the yeast with more ATP nor increase cell yield [], the mixture of galactose and cellobiose exhibited a synergistic effect on cell growth. Similar results have also been reported in the co-fermentation of cellobiose 29and xylose [23]. Since strong transcription usually caused declines in protein display efficiency [30], increasing the galactose concentration to 25 g/L decreased the fluorescence intensity to 2200, although the yeast grew much better with galactose than cellobiose.

Effects of inoculum density and miniscaffoldin II length on cellulosic ethanol production were then investigated. CMC (10 g/L) and galactose (20 g/L) were mixed as carbon source, and the yeast transformants without pre-induction were employed in single-step co-fermentation. After 6 days, the produced cellulosic ethanol was calculated based on the remaining galactose and the total ethanol generated. As shown in Fig. 5b, generally more cellulosic ethanol was produced by cells with a longer miniscaffoldin II, except for the higher inoculum density (A_600 nm_ = 0.50, 1.00, and 2.00) for which increasing CohII from 3 to 4 caused an obvious decrease in cellulosic ethanol production. Also, cellulosic ethanol production decreased sharply with increasing the inoculum density (A_600 nm_), probably because most galactose would be consumed before the expression of minicellulosomes was induced when more cells were present initially. The highest cellulosic ethanol produced from CMC reached 3.29 g/L (A_600 nm_ = 0.1, CohII = 4). For A_600 nm_ = 0.1 and CohII = 2, the cellulosic ethanol production was ~1.72 g/L, which was about 1.54-fold of that produced from CMC by the same strain pre-induced with galactose even at a much higher density of A_600 nm_ = 20 (Fig. 4c).

### Co-fermentation of cellulose and galactose for bioethanol production

**Fig. 6:**
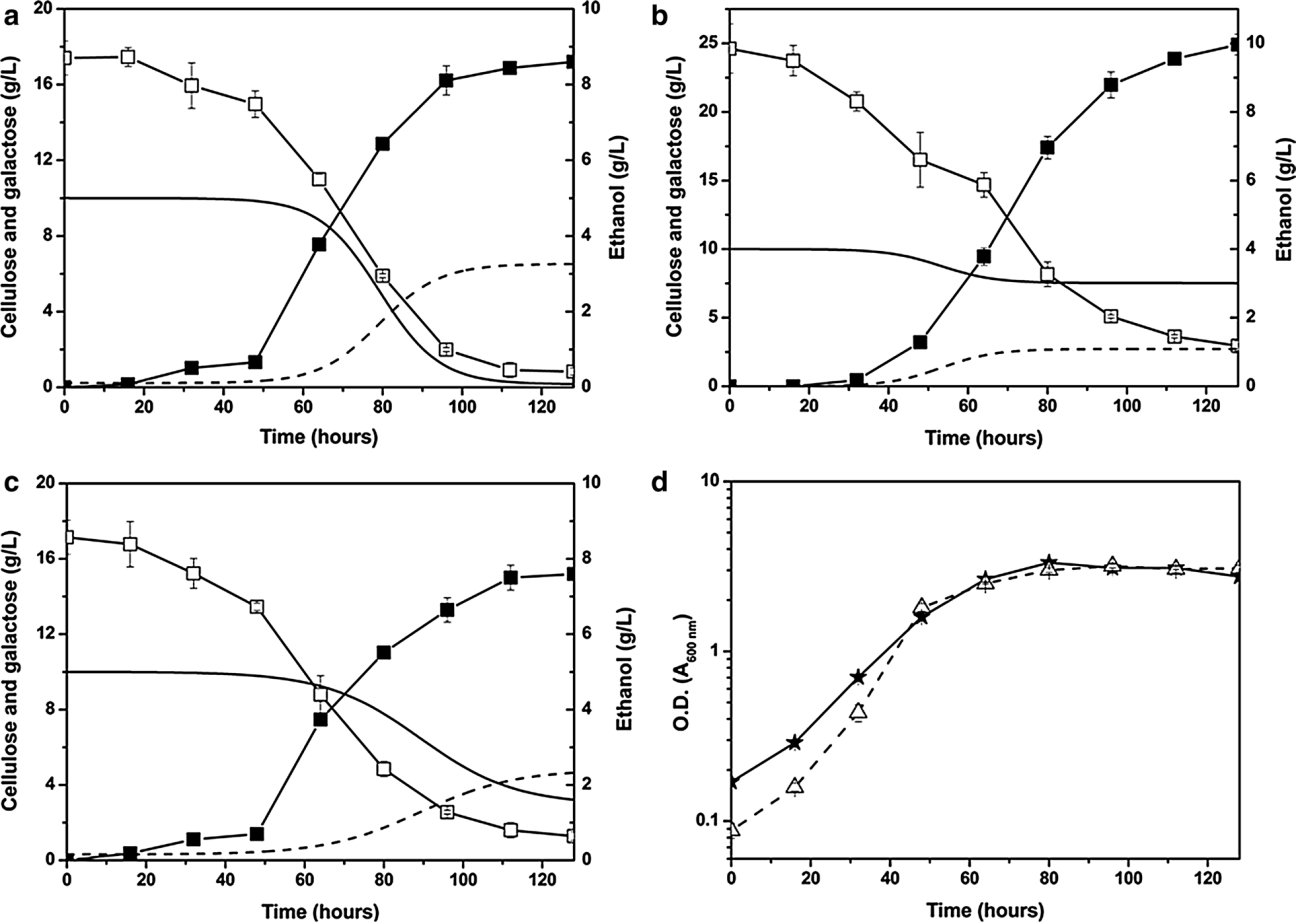
Kinetics of co-fermentation of galactose and CMC or PASC. **a** CMC-galactose mixture and cell-displayed minicellulosome (CohII = 4, *engy*, *cbhb, cdt*-*1*, *gh1*-*1*). **b** PASC-galactose mixture and cell-displayed minicellulosome (CohII = 4, *celcca* and *CA_C0911, cdt*-*1*, *gh1*-*1*). **c** CMC-galactose mixture and cell-free minicellulosome (docCipA was removed, CohII = 4, *engy*, *cbhb, cdt*-*1*, *gh1*-*1*). The cell inoculum density was A_600 nm_ = 0.10. Galactose (*solid* *line* with *open* *square*), total ethanol (*solid* *line* with *filled* *square*), cellulose (*solid line*), and cellulosic ethanol (*dash* *line*). **d** Growth kinetics of the fermentations shown in (**a**) (*solid line* with *filled* *star*), and (**c**) (*dash* *line* with *open* *triangle*)

Single-step co-fermentation of cellulose and galactose by the yeasts co-expressing the cellodextrin pathway (*cdt*-*1*, *gh1*-*1*) and bifunctional minicellulosome (CohII = 4) was studied (inoculated at A_600 nm_ = 0.10). Figure 6 shows the co-conversion of galactose and CMC with EBY100 (*cdt*-*1*, *gh1*-*1, engy, cbhb*) and PASC with EBY100 (*cdt*-*1*, *gh1*-*1, celcca*, *CA_C0911*). Cellulosic ethanol production in the co-fermentation was estimated from the total ethanol production and ethanol yield from galactose as the sole carbon source, which was found to be ~0.32 and ~0.44 g ethanol per gram of galactose for EBY100 (*cdt*-*1*, *gh1*-*1, engy, cbhb*) and EBY100 (*cdt*-*1*, *gh1*-*1, celcca*, *CA_C0911*), respectively (Additional file 1: Figure S6a, S6b). Similar galactose consumption curves in Fig. 6a and Additional file 1: Figure S6a, or Fig. 6b and Additional file 1: Figure S6b indicated that the presence of cellulose (CMC or PASC) did not seem to have significant effect on the conversion of galactose to ethanol by the engineered yeasts. Also, it has been reported that the *S. cerevisiae* grown on cellobiose or galactose gave almost the same ethanol yield [24]. Based on the assumption of the same ethanol yield from galactose and cellulose, cellulose consumption in the co-fermentation was also estimated. CMC-derived ethanol started to appear after 50 h, and reached its maximum level of 3.26 g/L 60 h later (Fig. 6a), with a specific productivity of ~62.61 mg cellulosic ethanol/g cell h. The total ethanol produced in the co-fermentation was 8.61 g/L, of which cellulosic ethanol accounted for ~37.9 %. CMC sharply decreased along with galactose during the period from 60 to 100 h. Only 0.17 g/L cellulose remained in the medium after 128 h, suggesting that more than 98 % of CMC has been hydrolyzed and used by the yeast. Similarly, PASC-galactose co-fermentation with EBY100 (*cdt*-*1*, *gh1*-*1, celcca*, *CA_C0911*) produced 9.97 g/L ethanol, in which 1.09 g/L (~10.9 %) was from PASC (Fig. 6b). PASC was mostly consumed during the period from 40 to 80 h, and ~25 % (2.5 g/L) was degraded. Apparently, PASC (amorphous cellulose) was more difficult to be degraded and used by the engineered yeasts than CMC (soluble cellulose). Nevertheless, the specific ethanol productivity from PASC still reached ~56.37 mg cellulosic ethanol/g cell·h, which was only ~10 % lower than that with CMC. However, co-fermentation of Avicel and galactose with the yeast EBY100 (*cdt*-*1*, *gh1*-*1*, *celccd*, *cbhb*) did not show any significant cellulosic ethanol production (data not shown).

To check if surface attachment of minicellulosomes on yeast cells would enhance cellulose utilization because of the enzyme-cell proximity effect [31], we removed the C-terminal docCipA from miniscaffoldin I* so that the produced minicellulosomes could not bind to miniscaffoldin II. As shown in Fig. 6c, the cellulosic ethanol produced from CMC was 2.33 and 3.21 g/L CMC still remained unused after 128 h. The corresponding specific productivity was ~33.34 mg cellulosic ethanol/g cell·h, a ~ 46.7 % decrease compared to yeasts with surface-displayed minicellulosomes, although both yeast strains had comparable growth in the co-fermentation (Fig. 6d).

## Discussion

Because *S. cerevisiae* does not have active cellodextrin transporters, the previously reported cellulose-utilization systems in cellulosome-engineered *S. cerevisiae* required extracellular β-glucosidase, and cellulose must be hydrolyzed into glucose before uptake. The major drawbacks of those systems for cellulosic ethanol production were: (i) Glucose inhibition on extracellular cellulases was unavoidable, resulting in inefficient cellulose hydrolysis. (ii) Carbon catabolite repression induced by glucose prohibited cells from co-utilizing other sugars. Here, we demonstrated the successful combination of a two-scaffoldin-based cellulosome with an intracellular cellodextrin pathway in *S. cerevisiae* to mimick the natural cellulose-utilization system in *C. thermocellum* and overcome the problems mentioned above. This cellulose-utilization system has never been reported for yeast.

In *S. cerevisiae*, galactose metabolic genes (GAL genes) are induced by the activator Gal4p in response to galactose but repressed by Mig1p when glucose is present. Even after GAL pathway induction, competitive transport of galactose and glucose is inevitable, because the majority of galactose is imported through the Gal2p transporter, which transports both galactose and glucose with high affinity [25]. In our work, galactose was not only an extra carbon source that could ensure cell division and proliferation, but also the inducer of strong GAL promoters for minicellulosome assembly. Yeast display of minicellulosomes usually took >40 h [9–12], thus only galactose could be metabolized at the beginning of the co-fermentation. After that, cellulose and galactose were simultaneously utilized without showing any sign of glucose repression, suggesting that the degradation of cellulose by the surface bifunctional minicellulosomes produced mostly cellodextrins, which also avoided glucose inhibition on displayed endo- and exo-glucanases. The generation of cellulosic ethanol suggested that the cellodextrins derived from cellulose were successfully taken up by cells and metabolized by intracellular cellodextrin pathway.

Interestingly, we also found that secretion using yeast α-factor facilitated the formation of protein complex on yeast cell surface, which has never been reported previously. This facilitation was weakened when a longer miniscaffoldin was used, probably because only a small fraction of proteins with α-factor could be secreted into the medium before binding to miniscaffoldin. On one hand, α-factor was able to provide more powerful force for the protein-scaffoldin complex to penetrate the cell membrane. On the other hand, fusion of α-factor (89 amino-acid residues) increased the steric hindrance for protein to bind to cohesin domains, especially for the longer miniscaffoldins.

The newly engineered *S. cerevisiae* was able to produce cellulosic ethanol at a high specific productivity (Table 1) far exceeding previously reported [9–12], and the fermentation process can be simplified to a single step, without requiring pre-induction and concentration of cells, which are required for other cellulosome-engineered yeasts. To accurately compare the cellulose utilization abilities of different yeasts, the specific ethanol productivities in Table 1 were calculated with the same calculation interval, which was from the time that cellulosic ethanol started to generate to the time that cellulosic ethanol reached its maximum level. More importantly, using galactose as a model, our work demonstrates a versatile yeast engineering strategy for co-utilization of cellulose with other biomass-derived sugars. This includes xylose, the most abundant pentose, and the second most abundant sugar next to glucose, found in biomass. Intracellular xylose conversion is only slightly affected by the presence or catabolism of intracellular glucose, but xylose transport can be strongly inhibited by glucose, which is the major reason hampering simultaneous fermentation of glucose and xylose [32, 33]. Theoretically, it is feasible to co-utilize cellulose and xylose by our yeast with changing the GAL promoters to constitutive promoters for miniscaffoldins expression and establishing a xylose assimilation pathway. Moreover, co-expression of lytic polysaccharide monooxygenases (LPMOs) and cellobiose dehydrogenases (CDHs) [34–36] with our cellulose-utilization system may further improve cellulose hydrolysis, especially for Avicel.

**Table 1:** Comparison of this work with other reports using cellulosome-engineered *S. cerevisiae* for cellulosic ethanol production

Pre-induction (galactose)	Added sugar for fermentation	Initial OD (A_600 nm_) for fermentation	cEtOH Concn (g/L)	Sp. *P*cEtOH (mg ethanol/g cell h)	References
20 g/L	10 g/L PASC	50	1.80	~1.67	[9]
20 g/L	10 g/L CMC	50	1.00	~0.68	[10]
20 g/L	10 g/L PASC	50	1.09	~0.74	[10]
20 g/L	10 g/L PASC	50	1.90	~1.72	[11]
20 g/L	10 g/L PASC	50	2.70	~1.83	[12]
No need	20 g/L galactose + 10 g/L CMC	0.1	3.26	~62.61	This work
No need	20 g/L galactose + 10 g/L PASC	0.1	1.09	~56.37	This work

Sp. *P*cEtOH denotes specific productivity of cellulosic ethanol (cEtOH). Pre-induction was carried out at 20 ^o^C for ~2 days. Cellulose-utilization systems: trifunctional minicellulosome without cellodextrin pathway [9–11], pentafunctional minicellulosome without cellodextrin pathway [12], bifunctional minicellulosome with cellodextrin pathway (this work)

## Conclusions

In summary, the cellulose-utilization systems from cellulosomal bacterium and cellulolytic fungus were combined and engineered into non-cellulolytic *S. cerevisiae*. The resulting yeasts succeeded in co-fermentation of cellulose and galactose, and showed an assimilating ability towards cellulose. Although galactose was used as a model in this work, this newly engineered system may be further applied for co-utilization of cellulose with other biomass-derived sugars, such as xylose.

## Additional file

10.1186/s13068-016-0554-6 Supplementary figures, tables and sequences. A file containing all the supplementary figures, tables and sequences referred to in the text.

## Abbreviations

CBP: consolidated bioprocessing
CMC: carboxymethyl cellulose
PASC: phosphoric acid-swollen cellulose
EG: endoglucanase
CBH: exoglucanase
BGL: β-glucosidase
GFP: green fluorescent protein
DSMZ: Deutsche Sammlung von Mikroorganismen und Zellkulturen
ATCC: American Type Culture Collection
LB: Luria–Bertani
IPTG: isopropyl-β-d-thiogalactopyranoside
YNB: yeast nitrogen base
SC: synthetic complete
PBS: phosphate-buffered saline
BSA: bovine serum albumin
α: enhancement coefficient
*F*_SU_: the GFP displayed on the EBY100 surface
*F*_IN_: the GFP localized in the cell cytoplasm
F*: the total fluorescence intensity of the yeast cells suspended in PBS (A_600 nm_ = 1) with enhancer treatment
F: the total fluorescence intensity of the yeast cells suspended in PBS (A_600 nm_ = 1) without enhancer treatment
DNS: 3, 5-dinitrosalicylic acid
α-factor: a yeast secretion signal
CohII: type II cohesin domain
ScaII: miniscaffoldin II
His-ScaII: His-tag-fused ScaII
His-EngY: His-tag-fused EngY
His-cbhB: His-tag-fused cbhB
LPMOs: lytic polysaccharide monooxygenases
CDHs: cellobiose dehydrogenases
