# Supplementary material for: Engineering yeast with bifunctional minicellulosome and cellodextrin pathway for co-utilization of cellulose-mixed sugars
Source: Biotechnol Biofuels. 2016 Jul 4;9:137. doi: 10.1186/s13068-016-0554-6 (PMC4932713; doi:10.1186/s13068-016-0554-6)
Supplement: Supplementary file 1 — 10.1186/s13068-016-0554-6 Supplementary figures, tables and sequences. A file containing all the supplementary figures, tables and sequences referred to in the text. [file 13068_2016_554_MOESM1_ESM.docx]

**Supplemental material**

**
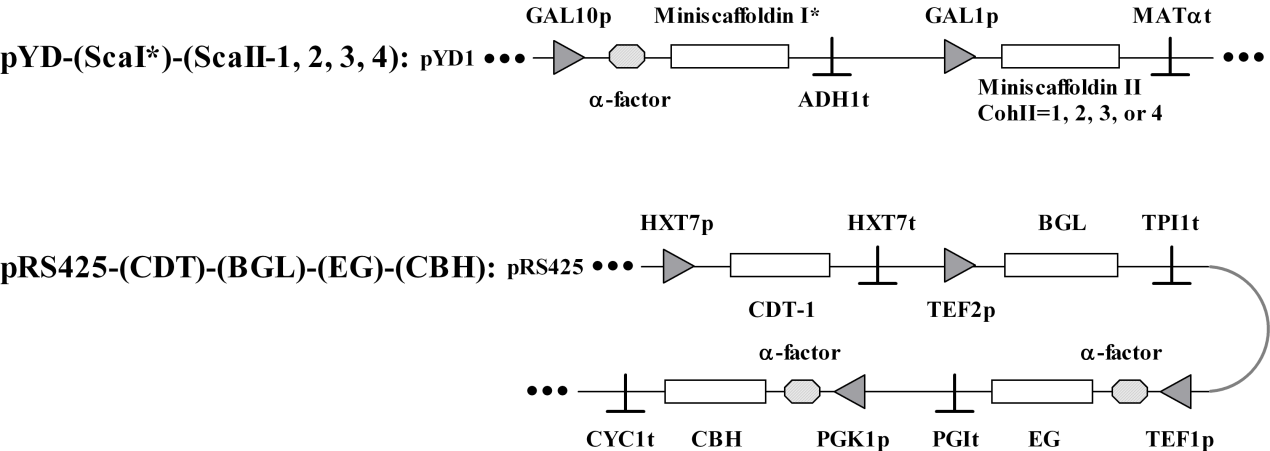
**

**Fig. S1** Structure of recombinant plasmids for cellulose-utilization system in *S. cerevisiae* EBY100. GAL1p and GAL10p are galactose-inducible promoters. HXT7p, TEF1p, TEF2p and PGK1p are constitutive promoters.


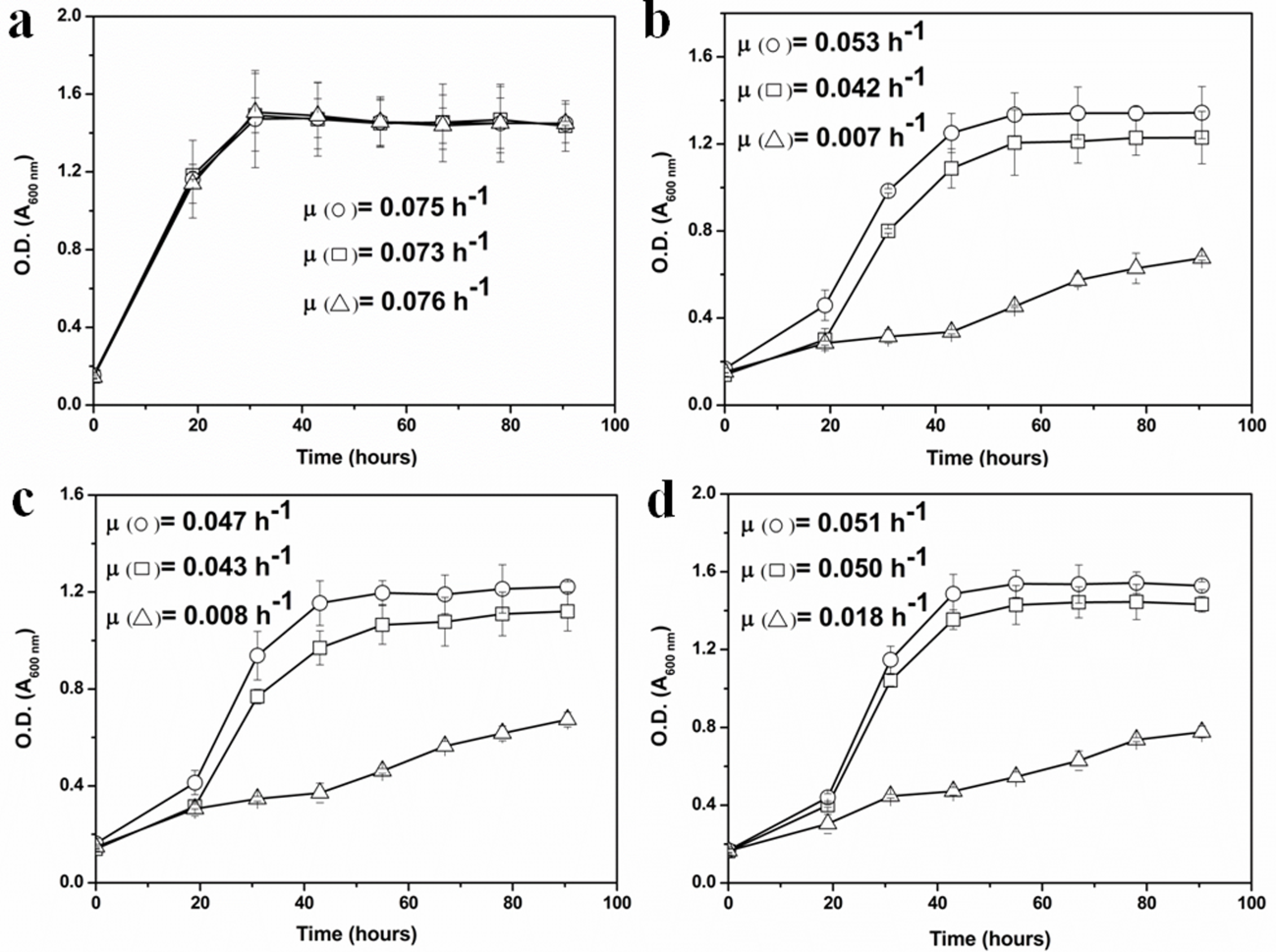


**Fig. S2** Functionality of the cellodextrin pathway engineered EBY100 with different β-glucosidases. 2% glucose (a), 1% cellobiose (b), 0.5% cellotriose (c), or 0.5% [cellotetrose](http://www.iciba.com/cellotetrose) (d) was used as the sole carbon source. CDT-1 and β-glucosidases did not have GFP tag. CDT-1 with GH1-1 (open circle), CDT-1 with BglA (open square), and CDT-1 with Ccel_2454 (open triangle).


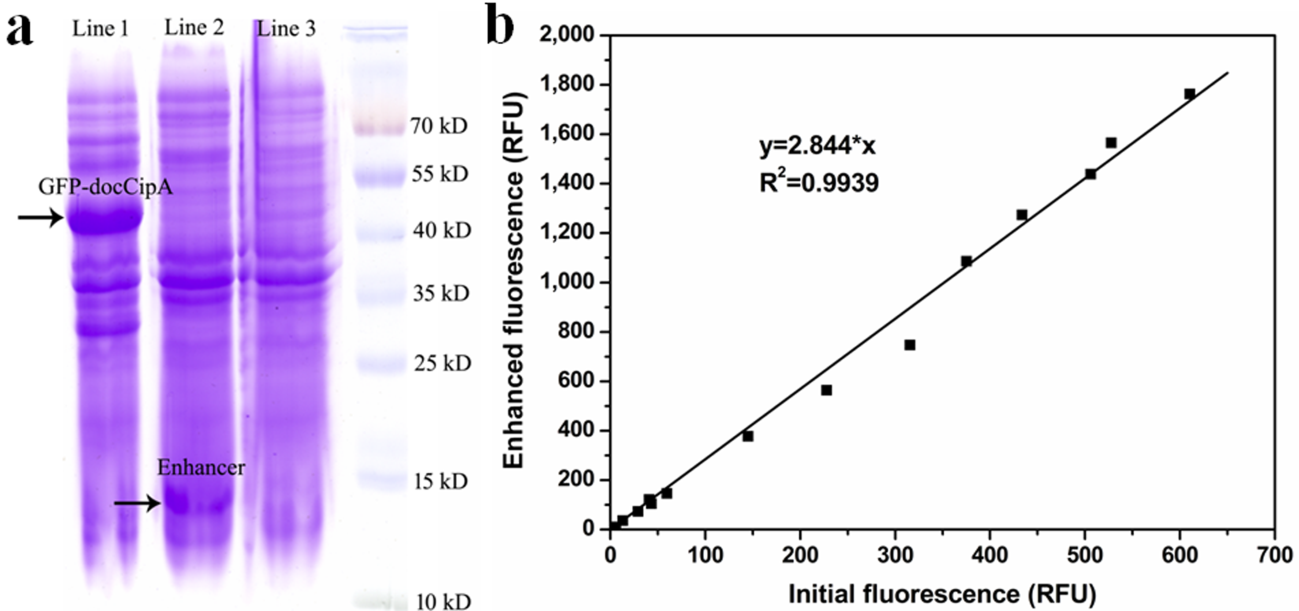


**Fig. S3** Fluorescence enhancement of GFP by Enhancer. SDS-PAGE of the GFP fusion (line 1) and Enhancer (line 2) that were produced in *E. coli* BL21 (DE3) (a). The control (line 3) was *E. coli* BL21 (DE3) harboring pET28a (+). C-terminus of GFP was fused with GS-docCipA. The enhancement coefficient (α) was 2.844 (b).


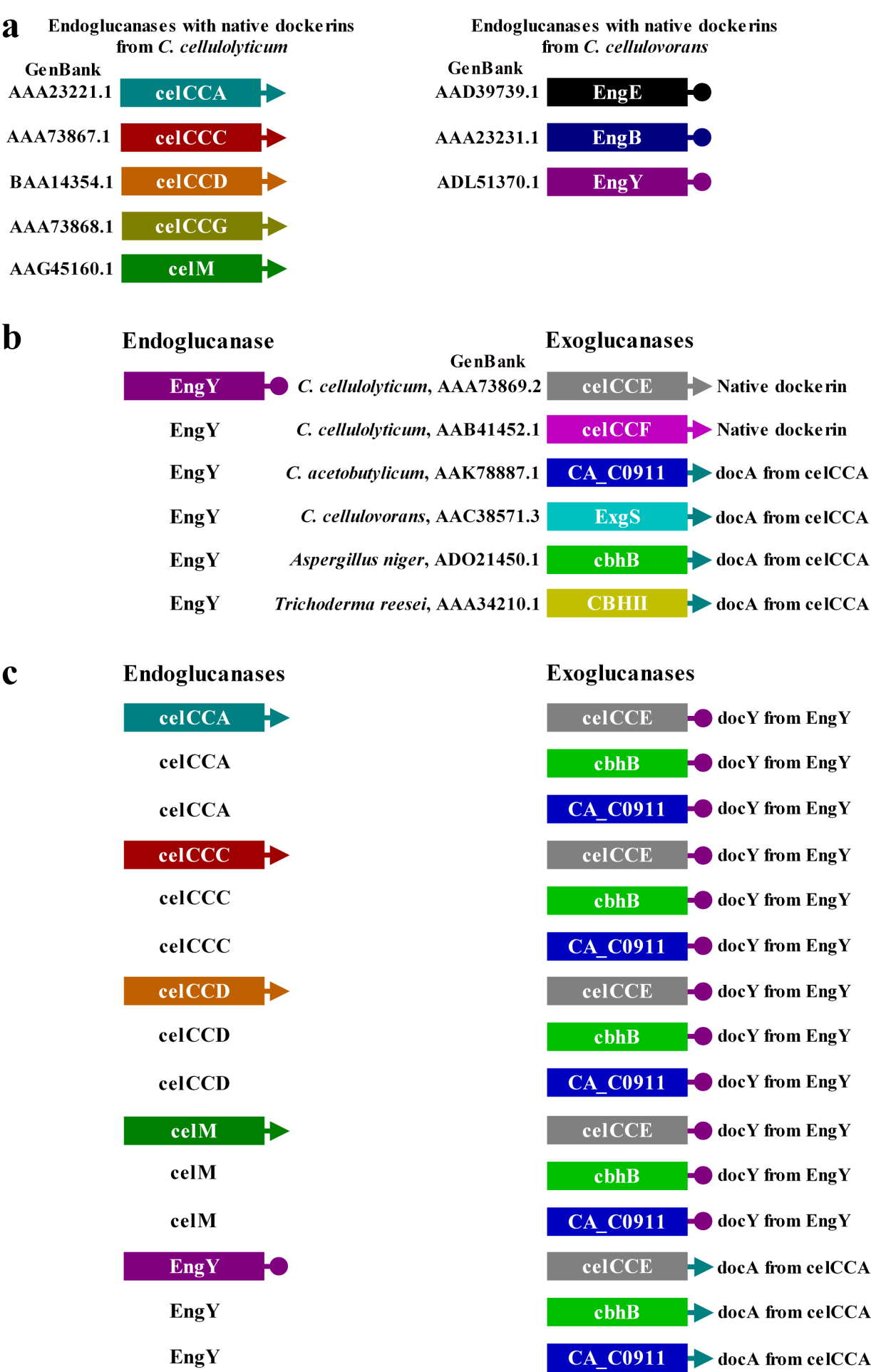


**Fig. S4** Native and recombinant endo- and exo-glucanases. Cellulases used for screening of endoglucanases (a), exoglucanases (b), and enzyme combinations (c).


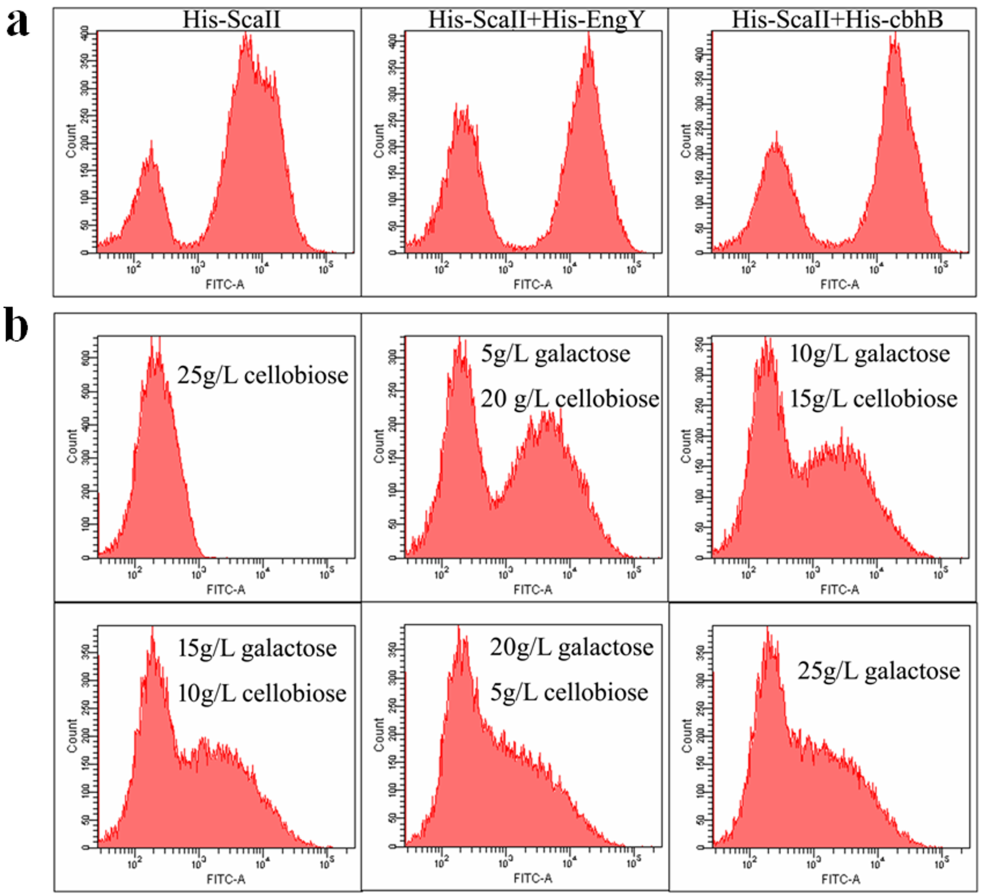


**Fig. S5** FACS analysis of the double-antibody stained EBY100 strains. Determination of the enzyme assembly efficiency (a), and effect of galactose on display of miniscaffoldin II (b). Miniscaffoldin II contained a C-terminal His-tag, while the His-tag at N-terminus of EngY or cbhB was selectively fused.


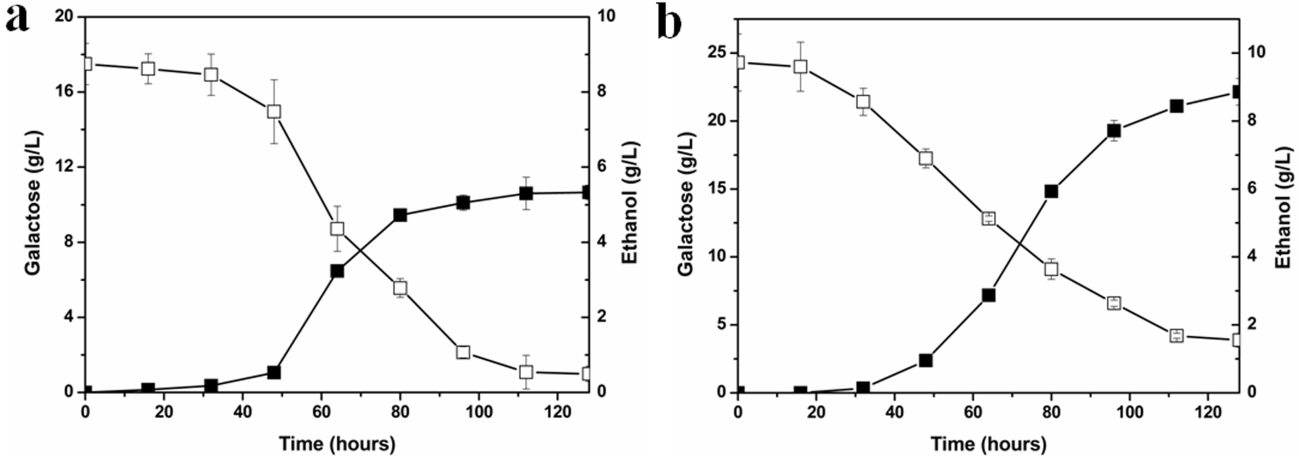


**Fig. S6** Fermentation with galactose as the sole carbon source by engineered yeasts. (a) EBY100 (*cdt-1*, *gh1-1*, *engy*, *cbhb*. CohII = 4); (b) EBY100 (*cdt-1*, *gh1-1*, *celcca*, *CA_C0911*, CohII = 4).

**Sequence of the codon-optimized *cbh2*:**

CAGGCATGTAGCTCTGTTTGGGGTCAATGTGGTGGTCAAAATTGGTCAGGCCCTACCTGTTGTGCTTCTGGCTCTACATGCGTTTATAGTAACGATTATTACTCTCAATGCTTGCCAGGTGCAGCTTCCAGTTCTTCATCCACAAGAGCAGCTTCTACTACTTCTAGAGTATCCCCAACTACTTCCCGTAGTTCTTCTGCTACACCTCCTCCAGGTTCTACTACTACTAGAGTCCCACCTGTTGGATCTGGTACTGCAACATACTCTGGCAACCCTTTCGTAGGTGTTACTCCATGGGCTAATGCTTACTATGCTAGTGAAGTATCCTCCCTAGCAATTCCATCTTTAACAGGAGCAATGGCTACAGCTGCTGCCGCTGTTGCTAAGGTCCCTTCTTTCATGTGGCTGGACACTCTTGATAAGACCCCACTAATGGAACAGACTCTAGCAGATATTAGAACTGCTAACAAGAATGGTGGTAACTATGCAGGTCAATTTGTCGTCTACGATCTTCCTGATAGAGATTGCGCCGCTTTAGCTAGTAATGGTGAATATTCAATTGCAGACGGTGGTGTTGCAAAGTACAAAAATTATATAGACACTATCCGTCAAATTGTTGTAGAATATAGTGACATTAGAACTTTGTTAGTTATAGAACCTGATTCTTTGGCAAATTTGGTCACTAATTTGGGTACACCAAAATGTGCTAATGCTCAATCAGCATATCTAGAATGTATTAACTACGCTGTAACACAACTGAATTTACCTAATGTTGCTATGTACTTGGACGCAGGACATGCAGGTTGGTTGGGTTGGCCAGCAAATCAAGATCCTGCTGCTCAGCTGTTTGCAAATGTCTATAAGAACGCTAGTAGTCCAAGAGCCCTTCGTGGTCTGGCAACAAACGTTGCTAACTACAACGGATGGAATATTACATCTCCTCCATCATACACACAAGGCAATGCTGTCTATAACGAAAAGTTGTATATCCATGCTATCGGTCCTTTGTTAGCCAATCATGGATGGAGTAACGCATTCTTTATTACCGACCAGGGAAGATCCGGTAAACAACCAACTGGACAACAACAGTGGGGTGATTGGTGTAACGTTATCGGCACAGGTTTTGGTATTCGTCCATCAGCTAACACTGGTGACTCTCTATTGGATTCTTTTGTATGGGTTAAACCTGGCGGCGAATGTGATGGTACTAGTGATTCCTCTGCCCCTCGTTTTGACTCACATTGTGCCTTGCCTGACGCTTTACAACCAGCCCCTCAAGCTGGCGCATGGTTCCAAGCTTACTTCGTACAACTATTAACCAACGCTAATCCTAGTTTCTTG

**Table S1** Parent vectors applied for gene cloning, fusion, and expression

| **Name** | **Description** | **Source/Reference** |
| --- | --- | --- |
| pET-28a(+) | Parent vector with T7 promoter and T7 terminator. | Novagen |
| pETDuet-1 | Parent vector with T7 promoter and T7 terminator. | Novagen |
| pYD1 | Parent vector for protein surface display on EBY100. | Invitrogen |
| pRS425 | Parent vector for protein intracellular expression in EBY100. | NEB |
| pRS424-HXT7p-GFP-HXT7t | Insert of HXT7 promoter, GFP, and HXT7 terminator in pRS424. | Prof. Huimin Zhao, University of Illinois at Urbana-Champaign |
| pET22-CohII-4 | Insert of miniscaffoldin II in pET22b (+) with CohII=4. | [1] |
| pYD-ScaI-ScaII-1, 2, 3, 4 | Insert of expression cassettes of miniscaffoldin I and II in pYD1 with CohII=1, 2, 3, or 4. | [1] |
| pUC19-PGK1p-F-CYC1t | Insert of PGK1 promoter, α-factor, and CYC1 terminator in pUC19. | [1] |
| pUC19-TEF1p-F-PGIt | Insert of TEF1 promoter, α-factor, and PGI terminator in pUC19. | [1] |
| pUC19-TEF2p-F-TPI1t | Insert of TEF2 promoter, α-factor, and TPI1 terminator in pUC19. | [1] |
| pUC19-GAL10p-F-ADH1t | Insert of GAL10 promoter, α-factor, and ADH1 terminator in pUC19. | [1] |
| pUC19-TEF2p-TPI1t | Insert of TEF2 promoter, and TPI1 terminator in pUC19. | This work |

**Table S2** Plasmids constructed for cloning of gene fusions

| **Name** | **Description** |
| --- | --- |
| pYD-CDT-GS-GFP | Construction of CDT-1-GS-GFP fusion. GS linker was fused to C-terminus of CDT-1. |
| pYD-GFP-GS-GH1 | Construction of GH1-1-GS-GFP fusion. GS linker was fused to N-terminus of GH1-1. |
| pYD-GS-docA | Construction of GS-docA fusion. |
| pYD-GS-docY | Construction of GS-docY fusion. |
| pYD-GFP-GS-docCipA | Construction of GFP-GS-docCipA fusion. GS linker was fused to C-terminus of GFP. |
| pUC19-TEF2-F-GFP-GS-docCipA-TPI1t | Construction of GFP-GS-docCipA expression cassette with α-factor. |
| pUC19-TEF2-GFP-GS-docCipA-TPI1t | Construction of GFP-GS-docCipA expression cassette without α-factor. |
| pRS424-HXT7p-CDT-HXT7t | Construction of CDT-1 expression cassette. |
| pRS424-HXT7p-CDT-GS-GFP-HXT7t | Construction of CDT-1-GS-GFP expression cassette. |
| pUC19-TEF2p-BGL-TPI1t | Construction of BGL expression cassettes. BGL was GH1-1, Ccel_2454, or BglA. |
| pUC19-TEF2p-GFP-GS-GH1-TPI1t | Construction of GFP-GS-GH1-1 expression cassette. |
| pUC19-TEF1p-F-EG-PGIt | Construction of EG expression cassettes. EG was celCCA, celCCC, celCCD, celCCG, celM, EngE, EngB, or EngY. |
| pUC19-TEF1p-F-HEngY-PGIt | Construction of N-terminal His-tag fused EngY expression cassette |
| pUC19-PGK1p-F-CBH-CYC1t | Construction of CBH expression cassettes. CBH was celCCE, or celCCF. |
| pUC19-PGK1p-F-CBH-GS-docA-CYC1t | Construction of CBH-GS-docA expression cassettes. CBH was CA_C0911, ExgS, cbhB, or CBHII. |
| pUC19-PGK1p-F-HcbhB-GS-docA-CYC1t | Construction of N-terminal His-tag fused cbhB-GS-docA expression cassette |
| pUC19-PGK1p-F-CBH-GS-docY-CYC1t | Construction of CBH-GS-docY expression cassettes. CBH was CA_C0911, or cbhB. |
| pUC19-GAL10p-F-ScaI*-ADH1t | Construction of miniscaffoldin I* expression cassette. Miniscaffoldin I* was truncated from miniscaffoldin I in pYD-ScaI-ScaII-1. |
| pUC19-GAL10p-F-ScaI**-ADH1t | Construction of miniscaffoldin I** (without dockerin domain docCipA) expression cassette. |
| pET22b-CohII-5, 6, 7, 8 | Construction of miniscaffoldin II with CohII=5, 6, 7, or 8 based on pET22-CohII-4. |

**Table S3** Plasmids constructed with genes, gene fusions (underlined) or gene cassettes (given in brackets) for expression

| **Name** | **Description** |
| --- | --- |
| pET28-Enhancer | Expression of Enhancer nanobody in *E. coli* BL21 (DE3). |
| pETDuet-GFP-GS-docCipA | Expression of GFP-GS-docCipA in *E. coli* BL21 (DE3). |
| pYD-(F-GFP-GS-docCipA)-(ScaII-1, 2, 3, 4, 5, 6, 7, 8) | Surface assembly of GFP on EBY100 (CohII=1, 2, 3, 4, 5, 6, 7, or 8) with α-factor. |
| pYD-(GFP-GS-docCipA)-(ScaII-1, 2, 3, 4, 5, 6, 7, 8) | Surface assembly of GFP on EBY100 (CohII=1, 2, 3, 4, 5, 6, 7, or 8) without α-factor. |
| pYD-(ScaI*)-(ScaII-1, 2, 3, 4) | Surface display of miniscaffoldin I* and II on EBY100 with CohII=1, 2, 3, or 4. |
| pYD-(ScaI**)-(ScaII-4) | Surface display of miniscaffoldin II (CohII=4), and secretion of the mutated miniscaffoldin I** |
| pRS425-(CDT) | Expression of CDT-1 in EBY100. |
| pRS425-(CDT-GS-GFP) | Expression of CDT-1-GS-GFP in EBY100. |
| pRS425-(BGL) | Expression of BGL in EBY100. BGL was GH1-1, Ccel_2454, or BglA. |
| pRS425-(GFP-GS-GH1) | Expression of GFP-GS-GH1 in EBY100. |
| pRS425-(CDT)-(BGL) | Expression of CDT-1 and BGL in EBY100. BGL was GH1-1, Ccel_2454, or BglA. |
| pRS425-(EG) | Expression of EG in EBY100. EG was celCCA, celCCC, celCCD, celCCG, celM, EngE, EngB, or EngY. |
| pRS425-(celCCE) | Expression of celCCE in EBY100 |
| pRS425-(EngY)-(CBH)/(CBH-GS-docA) | Expression of EngY and CBH in EBY100. CBH was celCCE, celCCF, CA_C0911, ExgS, cbhB, or CBHII. |
| pRS425-(CDT)-(GH1)-(EG)-(CBH)/(CBH  -GS-docA)/(CBH-GS-docY) | Expression of CDT-1, GH1-1, EG, and CBH in EBY100. EG was EngY, celCCA, celCCC, celCCD, or celM, while CBH was celCCE, CA_C0911, or cbhB. |
| pRS425-(CDT)-(GH1)-(HEngY)-(cbhB-  GS-docA) | Expression of CDT-1, GH1-1, His-tag fused EngY, and cbhB-GS-docA in EBY100. |
| pRS425-(CDT)-(GH1)-(EngY)-(HcbhB-  GS-docA) | Expression of CDT-1, GH1-1, EngY, and His-tag fused cbhB-GS-docA in EBY100. |

**Table S4** Primers used in this study

| **Name** | **Sequence** |
| --- | --- |
| Enhancer-F | CATGCCATGGCACAGGTGCAGCTGGTTGAAT |
| Enhancer-R | CCGCTCGAGTTAGTGATGATGATGGTGGTGG |
| CohII5-F | GCGTCGACGAAGCAACTCCAAGTATTGAAATGG |
| CohII5-R | CCCAAGCTTGTCTTTTAACGGTTCTGCCTCT |
| CohII6-F | CCCAAGCTTGAAGCAACTCCAAGTATTGAAATGG |
| CohII6-R | ATAAGAATGCGGCCGCGTCTTTTAACGGTTCTGCCTCT |
| CohII7-F | ATAAGAATGCGGCCGCAGAAGCAACTCCAAGTATTGAAATGG |
| CohII7-R | CCGCTCGAGGTCTTTTAACGGTTCTGCCTCT |
| CohII8-F | CCGCTCGAGGAAGCAACTCCAAGTATTGAAATGG |
| CohII8-R | Same as CohII7-R |
| Miniscaffoldin II-F | CTAGCTAGCATACATATGGAAGCAACTCCAA |
| Miniscaffoldin II-R | CTCAGTGGTGGTGGTGGTGGTGCTCGAG |
| GFP-HindIII-F | CCCAAGCTTATGGAATTCAGTAAAGGAGAAGAAC |
| GFP-HindIII-R | CCCAAGCTTTTTGTATAGTTCATCCATGCCATG |
| GFP-NheI-F | CTAGCTAGCATGAGTAAAGGAGAAGAACTTTTCACTG |
| GFP-XhoI-R | CCGCTCGAGCTATTTGTATAGTTCATCCATGCCATG |
| docCipA-F | CTAGCTAGCAATAAACCTGTAATAGAAGG |
| docCipA-R | CGCGGATCCTTACTGTGCGTCGTAA |
| GFP-GSdocCipA-F | CGGGATCCGATGAGTAAAGGAGAAGAACTTTTCACTG |
| GFP-GSdocCipA-R | CCGCTCGAGTTACTGTGCGTCGTAATCACTTG |
| GFP-GSdocCipA-F’ | CCGAGCTCCGATGAGTAAAGGAGAAGAACTTTTCACTG |
| GFP-GsdocCipA-R’ | GCTCTAGATTACTGTGCGTCGTAATCACTTG |
| pUC19-TEF2-F | CCGAGCTCGGTACCCGGGGAT |
| pUC19-TEF2-R | CCGAGCTCGTTTAGTTAATTATAGTTCGT |
| CDT-F | CGGAATTCATGTCGTCTCACGGCTCCCAT |
| CDT-R | CGGAATTCCTAAGCAACGATAGCTTCGGA |
| HXT7-CDT-F | CCCAAGCTTGTAAAACGACGGCCAGTGAGC |
| HXT7-CDT-R | CCCAAGCTTAATTAACCCTCACTAAAGGGAACA |
| Ccel_2454-F | CCGAGCTCATGCAATACGATCAGATAGA |
| Ccel_2454-R | GCTCTAGATCACAGAGCAAGAGCTAT |
| BglA-F | CCGAGCTCATGGAAAAGCTAAGATTTCCCAA |
| BglA-R | GCTCTAGATTACTTATTAGATCTTTCTATAAGCTCCTT |
| GH1-F | CCGAGCTCATGTCTCTTCCTAAGGATTTCCTCTG |
| GH1-R | GCTCTAGATTAGTCCTTCTTGATCAAAGAGTCA |
| GSGFP-overlap-F | GTCCGAAGCTATCGTTGCTAAGCTTCTGCAGGCTAGTG |
| GSGFP-overlap-R | CGGAATTCCTATTTGTATAGTTCATCCATGCCATG |
| CDT-overlap-F | Same as CDT-F |
| CDT-overlap-R | CACTAGCCTGCAGAAGCTTAGCAACGATAGCTTCGGAC |
| GFPGS-SacI-F | CGGAGCTCATGGAATTCAGTAAAGGAGAAGAAC |
| GFPGS-SacI-R | CGGAGCTCCTGTCCACCAGTCATGCTAGCA |
| ScaI*-F | CGGAGCTCCGAATGCAACACCGACCAAGG |
| ScaI*-R | TCCCCCGGGTTACTGTGCGTCGTAATCACTTGA |
| HisScaI*-F | CGGAGCTCCGCACCATCACCATCACCATAATGCAACACCGACC |
| HisScaI*-R | Same as ScaI*-R |
| ScaI**-F | Same as ScaI*-F |
| ScaI**-R | TCCCCCGGGTTAGATAGTTACTGTTCCTGGGTTAACTGC |
| P-Miniscaffoldin I-F | GGACTAGTCTGATTAATTACCCCAGAAATAAGGC |
| P-Miniscaffoldin I-R | GAAGATCTGCATGCCGGTAGAGGTGTGGTC |
| celCCA-F | CCGAGCTCCGTATGATGCTTCACTTATTCCGAATC |
| celCCA-R | ACGCGTCGACTTAGTTGCTTGGAAGCTTACTTACC |
| celCCC-F | CCGAGCTCCGGCTGATCAAATTCCTTTCCCA |
| celCCC-R | ACGCGTCGACCTAGTTAAGCAGTTTAACTTTTAGCTGAGC |
| celCCD-F | CCGAGCTCCGGCTATCAATTCTCAGGATATGGTAA |
| celCCD-R | ACGCGTCGACTTAGTCTCCAAGTAACATCATTTTCAAC |
| celCCG-F | CCGAGCTCCGGCAGGAACATATAACTATGGAGAAGC |
| celCCG-R | ACGCGTCGACTTAGCCTTGAGGTAATTGGGTG |
| celM-F | CCGAGCTCCGGCAGGAACACATGATTATTCAACTG |
| celM-R | ACGCGTCGACTTAACCTAAGATAGCCTTCTTTAAAAGAG |
| EngE-F | CCGAGCTCCGAATACTTTAGAATCAGTTGGCGGAG |
| EngE-R | ACGCGTCGACTTATATTGCTTTTTTTAAGAATGCAAGA |
| EngB-F | CCGAGCTCCGAAGACAGGTATTCGTGACATAACTTCTC |
| EngB-R | ACGCGTCGACTTAGCTTAAAAGCATTTTTTTAAGAACAG |
| EngY-F | CCGAGCTCCGGTAAATGCTGACACTACTGTCTCAAGA |
| EngY-R | ACGCGTCGACTTAGAAGCTATTAATTTGGCCTAGTA |
| HisEngY-F | CCGAGCTCCGCACCATCACCATCACCATGTAAATGCTGACACT |
| HisEngY-R | Same as EngY-R |
| cbhB-F | CCGAGCTCCGCAGCAGGTTGGCACCTACA |
| cbhB-R | GCTCTAGACAAACACTGCGAGTAGTACGCATT |
| HiscbhB-F | CCGAGCTCCGCACCATCACCATCACCATCAGCAGGTTGGCACC |
| HiscbhB-R | Same as chbB-R |
| CBHII-F | CCGAGCTCCGCAGGCATGTAGCTCTGTTTGG |
| CBHII-R | ACGCGTCGACCAAGAAACTAGGATTAGCGTTGG |
| celCCF-F | CCGAGCTCCGGCTTCAAGTCCTGCAAACAA |
| celCCF-R | ACGCGTCGACCTATTGGATAGAAAGAAGTGCTTTCTT |
| celCCE-F | TCCCCCGGGGATAGGACAAGCATTTGCCCT |
| celCCE-R | ACGCGTCGACTTACAGTGTGATTTTTCCTAACAAGA |
| celCCE-Non-F | Same as celCCE-F |
| celCCE-Non-R | ACGCGTCGACAGTTGGAGGAGTCACTGACCC |
| CA_C0911-F | CCGAGCTCCGGCAGCTGCTACAACTACAGATTCAT |
| CA_C0911-R | ACGCGTCGACATCTCCTGGTGTAGCTGTATTAGCGA |
| ExgS-F | CCGAGCTCCGTCAGCTGCACCAGTAGTGCCAAATAAT |
| ExgS-R | ACGCGTCGACACCTTTTAATCCTGGAGTAGGGTCT |
| docA-F | CTAGCTAGCGTAATTGTATATGGAGATTATAACAATGATGG |
| docA-R | CCGCTCGAGTTAGTTGCTTGGAAGCTTACTTACC |
| GSdocA-SalI-F | ACGCGTCGACAAGCTTCTGCAGGCTAGTG |
| GSdocA-SalI-R | Same as celCCA-R |
| GSdocA-XbaI-F | GCTCTAGAAAGCTTCTGCAGGCTAGTG |
| GSdocA-XbaI-R | GCTCTAGATTAGTTGCTTGGAAGCTTACTTACC |
| docY-F | CTAGCTAGCTTCATTTCAACCTTTGTTAACTCATC |
| docY-R | CCGCTCGAGTTAGAAGCTATTAATTTGGCCTAGTA |
| GSdocY-SalI-F | Same as GSdocA-SalI-F |
| GSdocY-SalI-R | Same as EngY-R |
| GSdocY-XbaI-F | Same as GSdocA-XbaI-F |
| GSdocY-XbaI-R | GCTCTAGATTAGAAGCTATTAATTTGGCCTAGTA |
| M3-SpeI-F | GGACTAGTGTAAAACGACGGCCAGT |
| RV-SpeI-R | GGACTAGTCAGGAAACAGCTATGAC |
| M3-SacII-F | TCCCCGCGGGTAAAACGACGGCCAGT |
| RV-SacII-BspEI-R | TCCCCGCGGTCCGGACAGGAAACAGCTATGAC |
| RV-SacII-BspDI-R | TCCCCGCGGATCGATCAGGAAACAGCTATGAC |
| M3-BspEI-F | TCCTCCGGAGTAAAACGACGGCCAGT |
| RV-BspEI-R | TCCTCCGGACAGGAAACAGCTATGAC |
| M3-BspDI-F | TCCATCGATGTAAAACGACGGCCAGT |
| RV-BspDI-R | TCCATCGATCAGGAAACAGCTATGAC |

**References**

1. Fan LH, Zhang ZJ, Yu XY, Xue YX, Tan TW. Self-surface assembly of cellulosomes with two miniscaffoldins on *Saccharomyces cerevisiae* for cellulosic ethanol production. Proc Natl Acad Sci USA. 2012;109:13260-13265.
